# Correlation between intraoral scanner and 3D confocal laser microscopy in early measurement of enamel loss due to dental erosion

**DOI:** 10.1186/s12903-025-07311-5

**Published:** 2025-12-04

**Authors:** Aya Y. Aly, Wegdan M. Abdel-Fattah, Mohamed A. Gepreel, Rania R. Afifi

**Affiliations:** 1 Faculty of Dentistry, Alexandria University, Alexandria, Egypt; 2 Egypt-Japan University of Science and Technology (E-JUST), Alexandria, Egypt

**Keywords:** Correlation, Intraoral scanner, Dental erosion, 3D confocal laser microscope

## Abstract

**Background:**

This study aimed to investigate the correlation between the intraoral scanners and 3D confocal laser microscopy in the early detection of dental erosion.

**Methods:**

Enamel samples (*n* = 36) with a thickness of 1 mm and dimensions of 3 × 4 mm were attached to the labial surfaces of sound -extracted anterior teeth. The specimens were scanned using an intraoral scanner (Carestream 3700) and a 3D confocal microscope (Keyence VK-X100) at baseline. After protection of the reference area, the erosive wear was created using 1% citric acid (ph.: 2.7) with continuous agitation and followed with brushing for 2 min. At time points (1, 3 and 6 h), follow-up scans were made and superimposed with the baseline scans to measure the erosion using the tools of the software of the intraoral scanner, each specimen was also measured under the 3D confocal laser microscope, where the height difference between the eroded and reference halves was analyzed. The values obtained from the intraoral scanner and the 3D confocal laser microscope were statistically analyzed.

**Results:**

Both methods could detect tissue loss after each acid immersion session, except for the intraoral scanner after the first hour of immersion; the loss values varied between both methods. The correlation was statistically significant at 6 h and a regression equation was obtained.

**Conclusion:**

An intraoral scanner was able to detect early dental erosion on flat enamel samples after 3 and 6 h of erosive acid challenge and the measurements obtained could be correlated to those obtained by the 3D confocal laser microscopy using the statistical equation.

## Background

Dental erosion is a disease of increasing prevalence worldwide due to the change of the dietary habits and the impact of some health conditions [1]. Early diagnosis of such condition is detrimental to avoid extremely advanced consequences such as aesthetic impairment and loss of the vertical dimension of occlusion [1]. Therefore, it is critical that oral health care clinicians have a better grasp of the methods used for the monitoring of the disease progression which influence the treatment options of each clinical situation [2]. 

The only evidence based technique for the detection and monitoring of dental erosion clinically is the application of indices, several index systems have been proposed but none of them have been universally accepted by the scientific community as the ‘gold standard’ [3]. Alternative methods have also been used such as the assessment of photographs or study models [4].

Clinical indices such as the Basic Erosive Wear Examination (BEWE) [5] and the Tooth Wear Evaluation System [6] have gained great acceptance worldwide because their application offers a feasible method to evaluate dental erosion. Clinical examination using indices may also be considered more sensitive to measure erosive wear progression, particularly in comparison to the evaluation of study casts [7]. 

However, these methods have several limitations such as standardization of the technique and obtaining semi quantitative values which makes monitoring early dental erosive wear in micrometers impossible [8]. Visual examination of dental tissues and application of index systems exhibit limited sensitivity, particularly in the early stages of mineral loss [7]. Typically, demineralization becomes clinically detectable only after substantial degradation of the hard dental tissues has occurred [7]. This diagnostic approach also lacks the capacity to quantify the extent of mineral loss over time, which presents a significant challenge in monitoring lesion progression and activity [7].

Meanwhile, intraoral scanners have emerged as a valuable tool for the early identification, measurement, and monitoring of tooth wear in a quantitative manner [9]. The integration of intraoral scanners has enabled the precise detection of minor enamel alterations, thereby improving clinicians’ ability to design evidence-based treatment strategies tailored to the specific progression and characteristics of each patient’s condition [9]. 

Recently the use of intraoral scanners has been suggested for the diagnosis of tooth wear taking benefit of their internal software in some intraoral scanner systems or using an external software that allows chair-side superimposition of 2 datasets [10–12], moreover, some in vivo studies have implemented the use of intraoral scanners in the monitoring of dental wear [13, 14].

Current literature lacks sufficient research on the capabilities and limitations of intraoral scanners in detecting and measuring early dental erosion. Moreover, there is a notable absence of comparative studies evaluating intraoral scanners’ performance against established gold standard technologies. A study assessed various intraoral scanners and software systems for detecting early dental erosion, finding that enamel loss measurements were consistently lower than those obtained via in vitro methods [11]. This tendency of intraoral scanners to underestimate erosion measurements has not been adequately correlated with conventional diagnostic methods, leaving clinicians without a reliable framework to compensate for these discrepancies in practice.

The 3D confocal laser microscopy is a technology that produces high-resolution 3D optical surface images, a laser beam is focused by an objective lens on the tested surface and the out-of-focus light is filtered out by confocal apertures to provide sharper images. Surface topography is obtained from consecutive images recorded in the x and y axes, combined with depth measured in the z axis. For the aim of quantitative measurement of surface loss at the micrometer scale the 3D laser microscopy could assess the tissue loss caused by erosion by comparing the affected surface to a reference [3]. 

Therefore, the aim of this study was to find the correlation between the measurements of the intraoral scanner system and the 3D confocal laser microscopy in the early detection of enamel loss due to dental erosion.

The hypothesis was that the measurements of the intraoral scanner could be correlated to those obtained by the 3D confocal laser microscope.

## Methods

### Sample size calculation

Sample size was estimated, assuming 5% alpha error and 80% study power. According to Witecy et al. [11], the mean (SD) enamel loss was 34.2 μm and 23.75 μm for non-contacting profilometry (PRO) and intraoral scanners (IOSi), respectively. The IOSi measurement can be ± 15 μm of the PRO measurement. Based on the difference between dependent means, the sample size was to be 35 samples, increased to 36 samples to make for processing errors.

Software: sample size was based on rosner’s method [15] calculated by G*Power 3.1.9.7 [16].

Human molars (*n* = 36) and anteriors (*n* = 36), extracted for therapeutic reasons, were collected from informed patients who agreed to voluntarily donate them for research, in compliance with the ethical standards of the Declaration of Helsinki. The teeth were examined to ensure they were free from caries, fractures, and restorations, cleaned using an ultrasonic cleaner and were kept hydrated throughout the experiment by being immersed in deionized water [12] in a temperature of 37℃ [17].

### Design of the specimens

The specimens were designed to be measured by both the intraoral scanner and the 3D confocal laser microscopy; the 3D confocal microscope required flat enamel samples that are perpendicular to its beam. On the other hand, the intraoral scanners have not proven the ability to precisely scan and measure the differences between subsequent scans of flat square-shaped samples; therefore, flat enamel samples were bonded to the labial surfaces of anterior teeth.

### Preparation of the enamel samples

Enamel samples were cut from sound molars as enamel is the normal substrate that suffers early minute tissue loss from exposure to different acids in the oral cavity. In order to prepare flat enamel samples (*n* = 36), then 1 mm thick longitudinal slices were cut using a microtome (Micracut 150, Metkon Metallography, Bursa, Turkey) to be with dimensions 4 × 3 mm [11].

Four grooves were made using a diamond stone (ISO 160/016, Komet, USA) on the midpoint of each limit of the enamel samples, to serve as reference points to highly standardize the test setup [11]. 

### Preparation of the anteriors

Anterior teeth were chosen because erosive wear was predominantly observed on the maxillary anterior teeth, particularly the incisors and canines, which are more susceptible due to their anatomical position and frequent exposure to dietary acids [18].

The labial surfaces of these anteriors were flattened at the center using a diamond bur (ISO 109/010, Komet, USA), the teeth were then mounted on a dimensionally stable silicone impression material (Zetaplus condensation silicone, Zhermack, Badia Polesine (RO) Italy) where the roots beyond the cement-enamel junction were completely imbedded within the silicone material.

The outer sides of the enamel samples and the flattened areas at the center of the anterior teeth were etched with 37% phosphoric acid etch (N Etch) for 30 s, cleaned with running distilled water and air dried. One layer of bonding agent (Tetric N universal bond) was then rubbed against the etched surfaces and light cured for 40 s.

**Fig. 1:**
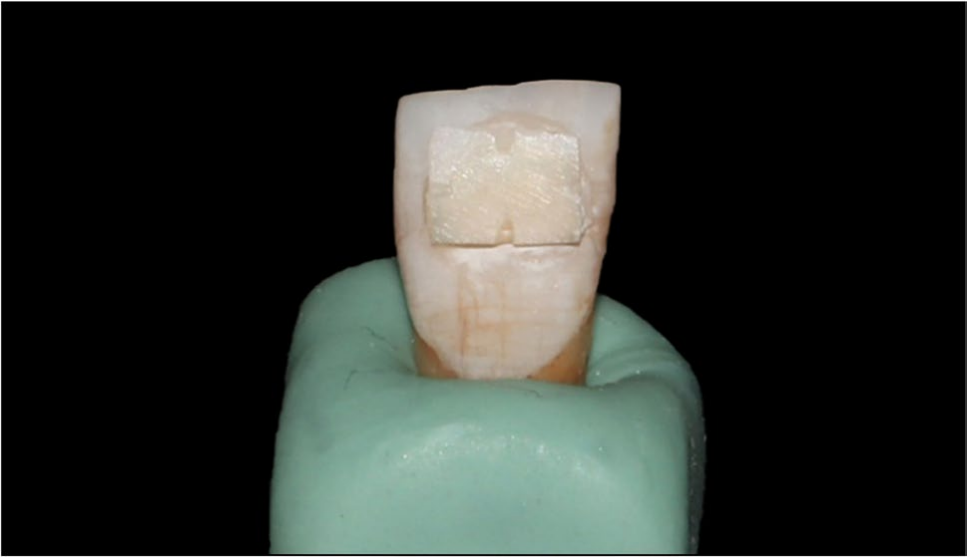
An enamel sample bonded to an anterior tooth

Each enamel sample was then bonded on the flattened area of each tooth with flowable composite (Tetric N Flow). Before curing of the composite, adjustments were made on the enamel samples while being placed under the 3D confocal laser microscope (KEYENCE VK-X100), to assure that the enamel sample was positioned perpendicular to it beam and then the composite was light cured for 40 s as shown in Fig. 1.

### Erosive tooth wear creation

Prior to the acid exposure (T0), all specimens were scanned with the intraoral scanner (CS 3700) and the 3D confocal laser microscope (KEYENCE VK-X100) to serve as baseline reference scans.

Each enamel sample was determined to be two halves along its upper and lower grooves, one half was assigned to be the reference area and protected from the erosive acid by applying an adhesive tape while the other half was determined to be exposed to the acid to develop erosive wear [7].

After applying the adhesive tape, the teeth were immersed in citric acid solution of concentration 1% and pH (pH = 2.7) with continuous agitation [12]. After 1 h (T1), 3 h (T2) and 6 h (T3) the teeth were removed out of the solution, rinsed for 20 s with deionized water and dried with compressed air for 5 s.The teeth were then brushed with an electrical tooth brush (Oral B Vitality Cross Action) for 2 min [12].

The aim of the experimental setup was the simulation of dental erosion conditions caused by extrinsic factors such as consumption of commonly used beverages that contain citric acid with the chosen pH and concentration [19]. The specimens were immersed in the acid for 1, 3 and 6 h to simulate the regular clinical visits at 1, 3 and 6 months [20].

Afterwards the adhesive tape was removed to perform the scanning and measuring procedures using the intraoral scanner and the 3D confocal laser microscopy.

### Measurement of dental erosion using the intraoral scanner

**Fig. 2:**
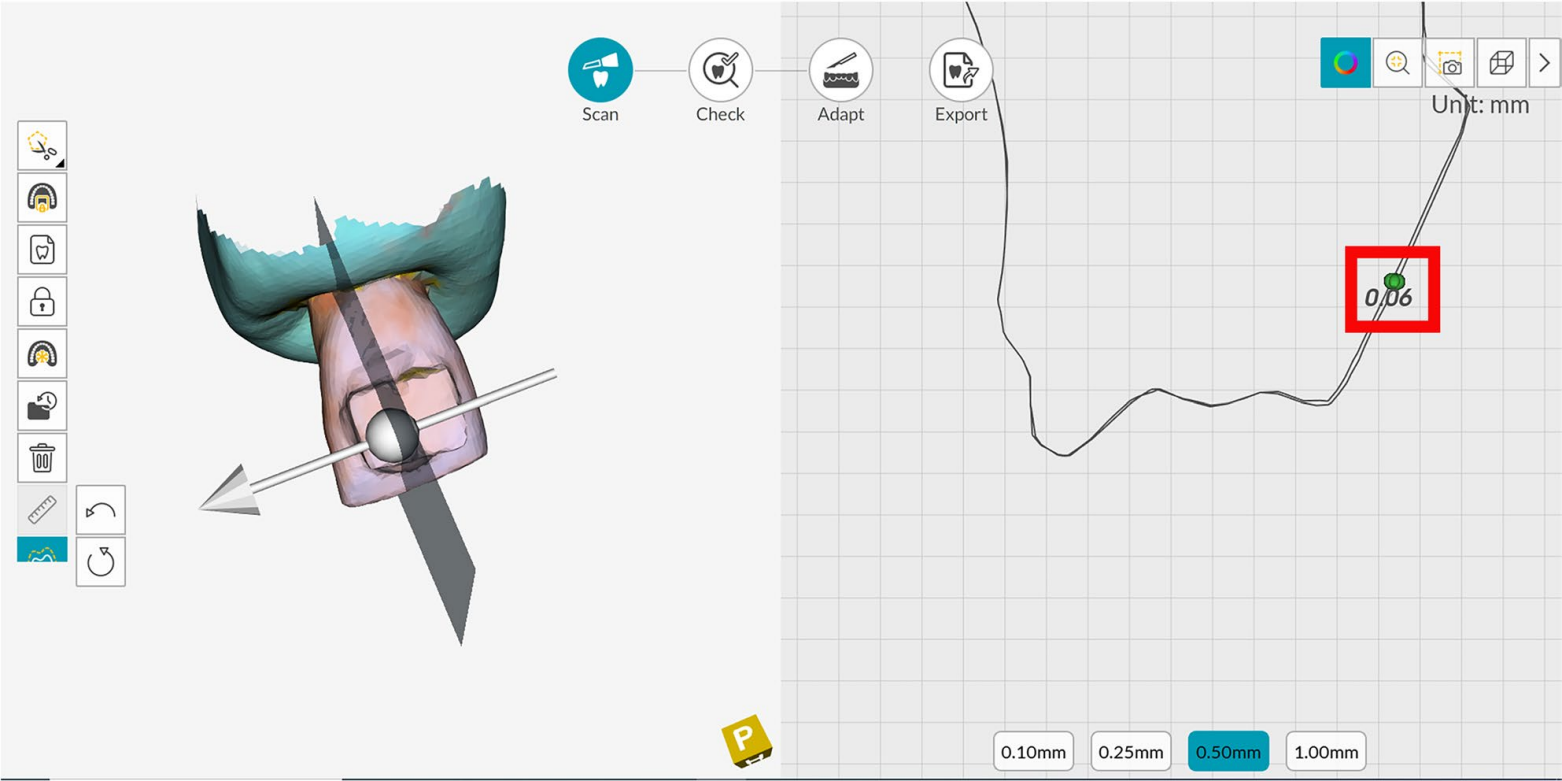
Superimposition of the baseline and follow up datasets. Left Panel: A 3D model of a tooth is shown with an alignment axis, likely used to standardize the measurement orientation between scans. Right Panel: A graph displays two virtual contours: Outer Line: Represents the baseline scan before erosion. Inner Line: Represents the follow-up scan after an erosive challenge. The red rectangle highlights the enamel loss, quantified as 0.06 mm, which is the vertical distance between the two lines at the point of maximum erosion

The whole surface of each specimen was scanned with the intraoral scanner (CS 3700). The intraoral scanner (CS 3700) was used in this study as it ranked first among 12 intraoral scanners in the level of trueness [21]. The internal software of the intraoral scanner (DEXIS IS ScanFlow) was used for the superimposition of the follow-up scan at (T1, T2 and T3) with the baseline scan at (T0) and measurements were made in the transition zone between the eroded area and the reference area of each sample using the assigned measuring tools in the software of the scanner to determine the dental erosion as shown in Fig. 2.

### Measurement of dental erosion using the 3D confocal laser microscope

Same specimens were also measured under the 3D confocal laser microscope (KEYENCE VK-X100) at the designated time point (T1, T2 and T3) where the transition zone between the eroded area and the reference area of the sample was situated at the center of the microscope’s field of view.

**Fig. 3:**
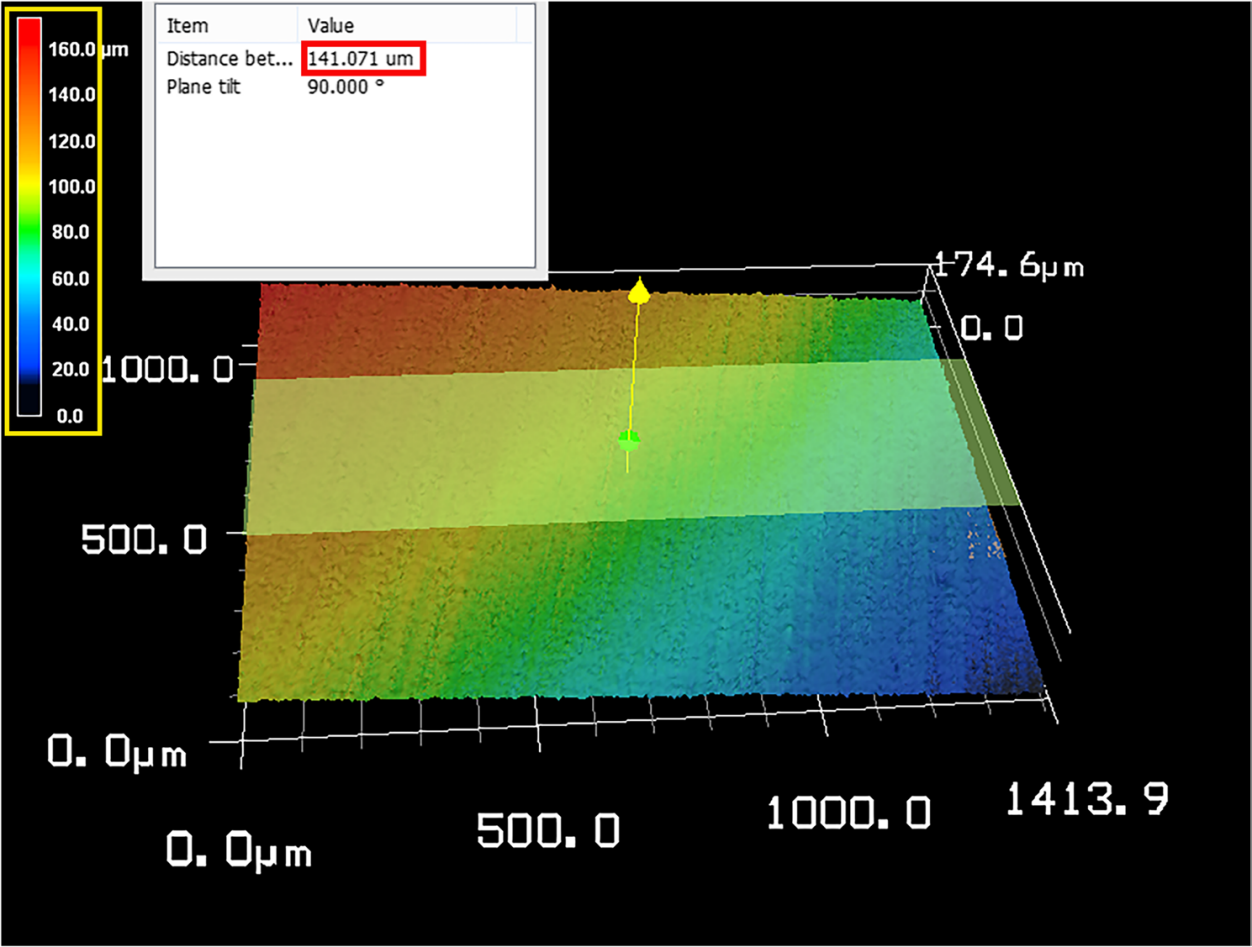
Measurement of dental erosion in microns between the reference area and the eroded area using 3D confocal laser microscopy, The yellow rectangle highlights the color scale, where: Red indicates the highest surface points (reference area) and Blue indicates the lowest points (eroded area). Red rectangle marks the height difference measured at plane tilt of 90°

The surface image of each sample was processed into a 3D image where the height difference between eroded and reference surfaces was measured in micrometers (µm) as shown in Fig. 3.

The 3D confocal laser microscopy was chosen as the standard method for comparison due to its scientifically proven ability to measure dental erosion quantitatively in in vitro studies without destruction of the tested samples [3, 22].

### Reproducibility

The reproducibility of both methods was tested prior to starting the experimental procedures. To simulate a comparable experimental procedure, one additional specimen was prepared as described before. After baseline scans, it was subjected to acidic challenge for 6 h and measurements were taken with the intraoral scanner and 3D confocal laser microscope and repeated 10 times. The results are demonstrated in (Table 1).

**Table 1:** Reproducibility of dental erosion values of the intra oral scanner and 3D confocal laser

	Intraoral Scanner (*n* = 10)	3D confocal Laser microscopy (*n* = 10)
Mean ± SD	68.00 ± 4.22	197.90 ± 0.74
95% CI	64.98, 71.02	197.37, 198.43

### Statistical analysis

Normality of variables were tested using Shapiro Wilk test and Q-Q plots. Non normal distribution was approved thus data were presented mainly using median, inter quartile range (IQR), minimum and maximum values in addition to mean and standard deviation. Comparison between intra oral scanner and 3D confocal laser microscope was done using Wilcoxon Sign Rank test while changes across time intervals were analyzed using Friedman test followed by post hoc test with Bonferroni correction. Agreement between readings obtained from both methods was assessed using Intra Class Correlation Coefficient (ICC), Bland Altman analysis and Spearman Rho correlation coefficient. All tests were two tailed and the significance level was set at *p* value ≤ 0.05. Data were analyzed using IBM SPSS, version 23, Armonk, NY, USA.

## Results

The statistical analysis of the values of erosive tissue loss measured by both testing methods from T1 to T3 were demonstrated (Table 2).

**Table 2:** Comparison of enamel loss (µm) between the intraoral scanner and 3D confocal laser microscopy at different time points

		Intraoral scanner (*n* = 36)	3D confocal laser microscopy (*n* = 36)	Test (*p* value)
T1	Mean ± SD	0.00 ± 0.00	67.53 ± 18.28	5.232 (< 0.0001*)
	Median (IQR)	0.00 (0.00)	69.50 (29.25)	
	Min – Max	0.00–0.00	33.00–98.00	
T2	Mean ± SD	34.17 ± 13.39	131.42 ± 24.81	5.232 (< 0.0001*)
	Median (IQR)	30.00 (20.00)	128.50 (42.50)	
	Min – Max	20.00–70.00	100.00–190.00	
T3	Mean ± SD	71.11 ± 26.70	209.72 ± 28.76	5.232 (< 0.0001*)
	Median (IQR)	65.00 (37.50)	209.50 (46.00)	
	Min – Max	40.00–160.00	170.00–272.00	
Test		72.00	72.00	
(*p* value)		(< 0.0001*)	(< 0.0001*)	
Pairwise comparisons		*p*_1_ < 0.0001*, *p*_2_ < 0.0001*, *p*_3_ < 0.0001*	*p*_1_ < 0.0001*, *p*_2_ < 0.0001*, *p*_3_ < 0.0001*	

*Statistically significant at *p* ≤ 0.05, *p*_1_: comparison between T1 and T2, *p*_2_: comparison between T1 and T3, *p*_3_: comparison between T2 and T3

**Fig. 4:**
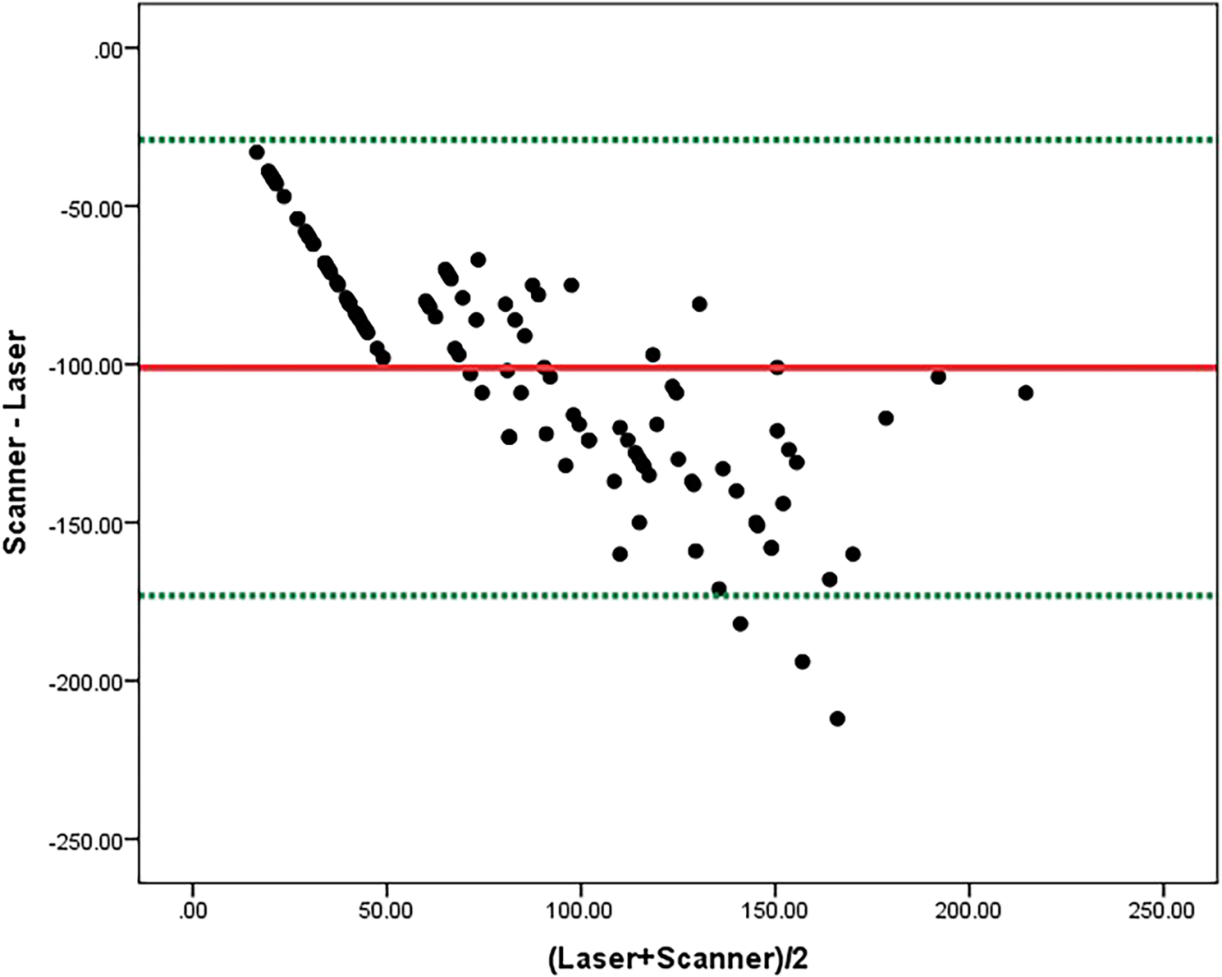
Bland Altman analysis between intraoral scanner and 3D confocal laser microscope

The reliability of both methods was evaluated using absolute agreement and the Intraclass Correlation Coefficient (ICC). The results were statistically significant (*p* < 0.0001), indicating a moderate positive correlation (ICC = 0.247) with a 95% confidence interval ranging from − 0.055 to 0.599. Bland–Altman analysis results are presented in Table 3 and illustrated in Fig. 4.

**Table 3:** Bland-Altman analysis evaluating the agreement of dental erosion values measured by the intraoral scanner and 3D confocal laser microscopy

	Bias (mean diff)	SD of bias	95% limits of agreement	*p* value
Intraoral scanner values vs. 3D confocal laser microscope values	−101.13	36.75	−173.16 – −29.1	< 0.0001*

*Statistically significant at *p* ≤ 0.05

**Fig. 5:**
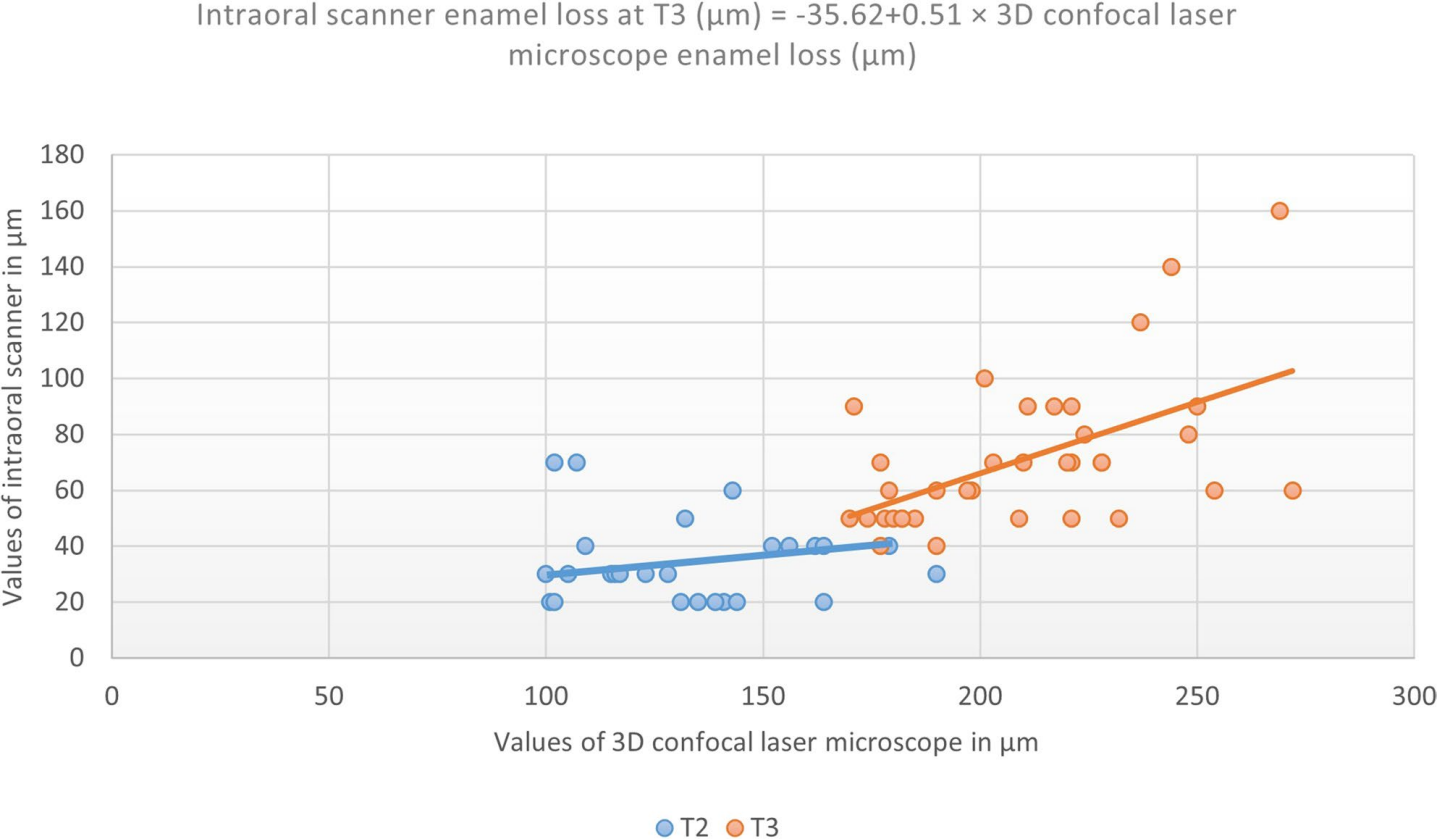
Spearman correlation between the values of intraoral scanner and 3D CONFOCAL LASER microscope at T2 and T3 showing regression lines and regression equation

Correlations between the intraoral scanner and the 3D confocal laser microscope measurements were assessed at time points (T1 to T3). At T1, no significant correlation was observed. Spearman correlation coefficients at T2 and T3 are presented in Table 4 and illustrated in Fig. 5.

At T3, a linear regression analysis yielded the following equation:

$$\begin{aligned}&\mathrm{Intraoral}\;\mathrm{scanner}\;\mathrm{enamel}\;\mathrm{loss}\;\mathrm{at}\;6\;\mathrm{hours}\;(\mu\mathrm m)\\&=\;-35.62\;+\;0.51\;\times\;3\mathrm D\;\mathrm{confocal}\;\mathrm{laser}\\&\mathrm{microscope}\;\mathrm{enamel}\;\mathrm{loss}\;(\mu\mathrm m)\end{aligned}$$

**Table 4:** Correlation of dental erosion (µm) measured via the intraoral scanner and 3D confocal laser microscopy at 3 and 6 h

	*r*_s_ (Spearman Rho correlation coefficient)	*p*-value
Correlation at 3 h	0.204	0.233
Correlation at 6 h	0.518	0.001*

p*: *p* value is significant at *p* ≤ 0.05

## Discussion

The primary aim of this study was to investigate the correlation between the measurements of intraoral scanners and the 3D confocal laser microscopy in the early detection of dental erosion. The hypothesis of this study was partially accepted as the results revealed that the intraoral scanner was able to detect dental erosion at 3 and 6 h; however, it failed to detect tissue loss at 1 h and the correlation was only calculated at the 6- hour time point.

These findings are consistent with those reported by Machado et al. [23], who demonstrated that intraoral scanners have potential clinical utility in detecting and quantifying early and advanced erosive tooth wear. The progressive improvement in scanner performance over time in the present study supports the notion that intraoral scanners may be more effective in identifying cumulative enamel loss rather than subtle, early-stage changes.

In this study, erosive wear loss of 100 μm—measured using 3D confocal laser microscopy—was detectable by the intraoral scanner. Although this value exceeds those typically reported by in vitro methods, it remains significantly below the threshold detectable by the human eye. Since the human eye can only resolve depth differences of approximately 200 microns [24], relying solely on visual depth assessment is not a reliable method for detecting wear within a practical diagnostic timeframe. Notably, advanced in vitro quantitative methods have demonstrated that average wear over a six-month period is approximately 25 microns [25]. Therefore, it’s essential to consider the limitations of each assessment method considering this constraint.

The inability of the intraoral scanner to detect enamel loss at T1 aligns with the findings of Witecy et al. [11], who reported similar limitations when comparing the Carestream 3600 intraoral scanner to a non-contact profilometer. In their study, the scanner failed to identify erosion following the initial cycle of acid etching. This limitation may be attributed to the scanner’s insufficient sensitivity for detecting minimal tissue loss in the micrometer range, which is critical for monitoring early stages of dental erosion.

In the present study, the mean enamel loss measured by the 3D confocal laser microscope after one hour of acid immersion was 67.53 μm. This value likely falls within the measurement uncertainty range of intraoral scanners, rendering early detection unreliable. This assumption is further supported by O’Toole et al. [7], who noted that current intraoral scanner software cannot accurately detect tissue loss below 50 μm due to inherent flaws in scan registration algorithms.

Although the use of Spearman correlation is methodologically acceptable, the correlation analysis does not account for bias or systematic error. Consequently, intraclass correlation coefficient (ICC) and Bland–Altman plots were applied for their suitability for evaluating agreement between measurement methods. The intraclass correlation coefficient (ICC) was statistically derived from measurements obtained using both the intraoral scanner and the 3D confocal laser microscope at three time points (T1, T2, and T3). At T1, the intraoral scanner failed to detect any erosion, resulting in zero values across all measurements. This limitation contributed to weak agreement between the two methods.

Moreover, it was observed that the intraoral scanner consistently underestimated erosive wear compared to the 3D confocal laser microscope, as confirmed by the mean bias in the Bland–Altman analysis and previously reported by Witecy et al. [11] and O’Toole et al. [7]. This could be attributed to the mechanisms of registration and analysis in the software of most intraoral scanners which use registration algorithms that minimize the distance between similar areas in subsequent scans into the closest possible approximation. This results in positive data (tissue gain), which leads to a decrease in the value of erosion [10, 26]. 

To address this limitation, our study aimed to establish a correlation between the values of the intraoral scanner and the 3D confocal laser microscope to detect and monitor the early erosive dental wear, which was verified after 6 h, the correlation observed at the 6-hour time point may help compensate for the scanner’s underestimation of erosion by applying the regression equation.

Given that, existing literature [27–29] concluded that an 18-month interval is likely required to reliably detect changes using clinical indices as accurate detection of early erosive wear typically involves assessing changes in depth, surface texture, translucency, and color [7]. Nevertheless, in this study, measurements obtained by the intraoral scanner at T3 (corresponding to six months of clinical progression) showed a correlation with those recorded using 3D confocal laser microscopy. This correlation supports the effectiveness of the intraoral scanner in detecting early stages of dental erosion in comparison to the traditional clinical indices. The correlation analysis highlights a critical shift in dental erosion diagnostics from traditional visual methods to advanced digital technologies.

Ultimately, obtaining an equation supported by statistical analysis to convert intraoral scanners’ values to values that can be obtained by advanced laboratory equipment, such as 3D confocal laser microscopy, at the regular 6-months follow-up visits represents a significant advancement in the context of digitalization of tooth wear diagnosis.

The limitations include that the study was conducted using in vitro samples, which may not fully replicate the complexities of clinical conditions, such as the presence of saliva, patient movement, and optical noise. Although the sample size was adequate for comparing methodologies, it may not reflect the full spectrum of variability encountered in clinical populations. Additionally, the study focused solely on a single erosive challenge and material—human enamel—limiting the generalizability of the findings to other materials or clinical scenarios.

## Conclusions

In a simulated clinical setting, the intraoral scanner was able to detect early dental erosion on flat enamel samples after 3- and 6-hour erosive acid exposures. A positive correlation was statistically approved when the values measured by the intraoral scanner were compared with the tissue loss values obtained from 3D confocal laser microscopy, and the regression equation was calculated from the 6-hour data. Although intraoral scanners show potential for early erosion detection, their accuracy remains lower than that of 3D confocal laser microscopy. Their primary value may lie in tracking changes over time, supporting preventive care and patient education, though further in vivo validation is needed.

## Data Availability

All the data generated or analyzed during this study are included in this article. Further inquiries can be directed to the corresponding author.

## Abbreviations

T1: 1 h of immersion in acid
T2: 3 h of immersion in acid
T3: 6 h of immersion in acid
